# Mango Fructokinases Inhibit Sugar Accumulation and Enhance Energy Metabolism in Transgenic Tomato

**DOI:** 10.3390/plants14223526

**Published:** 2025-11-19

**Authors:** Bin Zheng, Songbiao Wang, Hongxia Wu, Xiaowei Ma, Wentian Xu, Kunliang Xie, Meng Gao, Yanan Wang, Chengming Yan, Zixin Meng, Li Li

**Affiliations:** 1 Key Laboratory of Tropical Fruit Biology, Ministry of Agriculture and Rural Affairs, Key Laboratory of Postharvest Physiology and Technology of Tropical Horticultural Products of Hainan Province, South Subtropical Crops Research Institute, Chinese Academy of Tropical Agricultural Sciences, Zhanjiang 524091, China; zhengbin@catas.cn (B.Z.);; 2 Horticulture Department, Nanjing Agricultural University, Nanjing 210095, China; 3 National Key Laboratory for Tropical Crop Breeding, Sanya Research Institute of Chinese Academy of Tropical Agricultural Sciences, Sanya 572024, China; 4 Guangxi Engineering Research Center of Green and Efficient Development for Mango Industry, Guangxi Subtropical Crops Research Institute, Guangxi Academy of Agricultural Sciences, Nanning 530001, China

**Keywords:** mango (*Mangifera indica* L.), FRK, sugar, genes

## Abstract

Sugar content critically determines mango fruit quality and varies significantly among varieties. Preliminary studies indicate that *fructokinases* (*MiFRKs*) *MiFRK1* and *MiFRK2* likely regulate intervarietal sugar variation. We characterized these *MiFRKs* using heterologous expression in tomato. Both isoforms phosphorylate fructose, promoting downstream catabolism, with *R-MiFRK2* (from low-sugar ‘Renong No. 1’) exhibiting higher activity than *T-MiFRK2* (high-sugar ‘Tainong No. 1’) and *MiFRK1*. Transcriptomic and metabolic analyses reveal that *MiFRK* overexpression inhibits sugar accumulation by altering the expression of key metabolic genes, including sucrose degradation enzymes (*invertases*), starch breakdown genes (*β-amylases*), and glycolytic genes (*enolases*). Intriguingly, *MiFRK1* and *MiFRK2* exhibit distinct regulatory effects on these pathways, suggesting functional specialization between the two isoforms. These findings provide novel insights into the molecular mechanisms through which *MiFRKs* govern sugar metabolism in mango, highlighting their potential as key targets for metabolic engineering to enhance fruit quality.

## 1. Introduction

Sweetness is a key quality trait in fleshy fruits, which is primarily determined by their soluble sugar content. These sugars serve three critical physiological functions: providing metabolic substrates for energy production and carbon skeletons, maintaining osmotic potential for cellular expansion through turgor pressure regulation, and functioning as signaling molecules that orchestrate developmental processes and mediate stress responses [1,2]. In fruit crops, sucrose, sorbitol, and occasionally trehalose or raffinose are synthesized in leaves (source organs) and transported to sink tissues such as fruits, tubers, and seeds [2]. Notably, apple (*Malus domestica*) predominantly transports sorbitol and sucrose [3], while citrus varieties mainly utilize sucrose as the primary translocated sugar [4].

The metabolic conversion of transported photosynthates in developing fruits primarily yields hexose sugars, particularly fructose and glucose, which form the biochemical basis for assimilate partitioning in sink tissues. In higher plants, this process is tightly regulated by two key enzymes: *FRK* and *hexokinase* (HXK). These enzymes serve as crucial gatekeepers of hexose phosphorylation and subsequent metabolic flux [5]. Although both enzymes exhibit fructose phosphorylation capacity, *FRK* demonstrates superior substrate specificity and catalytic efficiency for fructose compared to *HXK* [6,7]. As the principal fructose-phosphorylating enzyme, *FRK* catalyzes the irreversible conversion of fructose to fructose-6-phosphate (F6P), thereby establishing F6P as a central metabolic node in sink tissues [8].

Extensive functional characterization of *FRKs* across diverse fruit species has revealed their pivotal role in sugar metabolism regulation. Studies in satsuma mandarin (*Citrus unshiu*) [9], apple, and Japanese pear (*Pyrus pyrifolia*) [10] consistently demonstrate an inverse relationship between FRK activity decline and fructose accumulation during fruit maturation. This phenomenon reflects a conserved expression pattern where *FRK* genes are predominantly expressed during early fruit development but downregulated during ripening phases. Such expression dynamics have been documented in multiple species, including *EjFRK* in loquat (*Eriobotrya japonica*) [11], *MrFRK2* in Chinese bayberry (*Myrica rubra*) [12], *LbFRK7* in wolfberry (*Lycium barbarum*) [13], *HpFRK1* in red-fleshed pitaya (*Hylocereus undatus*) [14], *McFRK2* in noni (*Morinda citrifolia*) [15], *FaFRK3* in strawberry (*Fragaria ananassa*) [16], and *MdFRK2* in apple [17].

Functional studies reveal that *FRKs* can differentially regulate sugar metabolism. Overexpression of *MdFRK2* [18] and *PbFRK1* [19] in tomato (*Solanum lycopersicum*) enhances FRK activity while reducing fructose, glucose, and sucrose levels, suggesting a role in sugar catabolism. Conversely, *PpyFRK5* in pear exhibits a positive correlation with sucrose accumulation. Transient overexpression of *PpyFRK5* significantly elevates the sucrose content, where its overexpression elevates while silencing diminishes the sucrose content, demonstrating its promotive role in sugar metabolism [20].

Beyond their metabolic roles, *FRKs* also play critical roles in organogenesis and stress responses. In tomato, *SlFRK1* and *SlFRK2* not only regulate flowering time but also significantly influence root architecture, stem elongation, and seed development [21]. Notably, *LeFRK2* is essential for phloem and xylem differentiation, facilitating sugar and water transport [22]. In apple, *MdFRK2* exhibits remarkable functional plasticity in abiotic stress responses, including its well-characterized role in salinity tolerance [23]. Notably, under drought conditions, transgenic overexpression of *MdFRK2* promotes root system development through coordinated regulation of carbohydrate metabolism, hormone signaling, and osmotic adjustment, thereby improving drought resistance [24].

Mango (*Mangifera indica* L.), widely acclaimed as the ‘King of Tropical Fruits’, is particularly valued for its distinctive aroma and characteristically sweet, succulent flesh, with sweetness constituting a fundamental quality determinant in mango fruits. Its sugar accumulation pattern follows the typical model of early-stage starch accumulation followed by subsequent starch degradation into soluble sugars [25]. The predominant soluble sugars in ripe mango fruit are sucrose, fructose, and glucose [26]. In our previous study, we identified two *MiFRKs* that may be involved in regulating the differential fructose content between the low-sugar mango cultivar ‘Renong No. 1’ and the high-sugar cultivar ‘Tainong No. 1’ [27]. To further investigate their functions, this study conducted genetic transformation of these two *MiFRKs* in tomato and analyzed the physiological characteristics and gene expression profiles of the transgenic tomato plants. The aim was to elucidate their roles in mango sugar metabolism and provide a theoretical basis for the foundation regulation of fruit sweetness.

## 2. Results

### 2.1. Comparative Analysis of MiFRK1 and MiFRK2 Genes in Two Mango Varieties

In our previous studies [27], we successfully amplified the coding DNA sequences (CDSs) of *MiFRK1* and *MiFRK2* from the fruit pulp cDNA of two mango cultivars, ‘Renong No. 1’ and ‘Tainong No. 1’. Notably, the *MiFRK1* gene exhibited complete sequence identity between the two cultivars and was thus uniformly designated *MiFRK1* (Figure S1). In contrast, the *MiFRK2* sequences displayed 12 nucleotide polymorphisms within the coding region, resulting in amino acid variations at positions 33, 87, 139, 252, 277, 293, and 317 (Figures S2 and S3). Consequently, the two variants were designated *R-MiFRK2* (from ‘Renong No. 1’) and *T-MiFRK2* (from ‘Tainong No. 1’).

**Figure 1:**
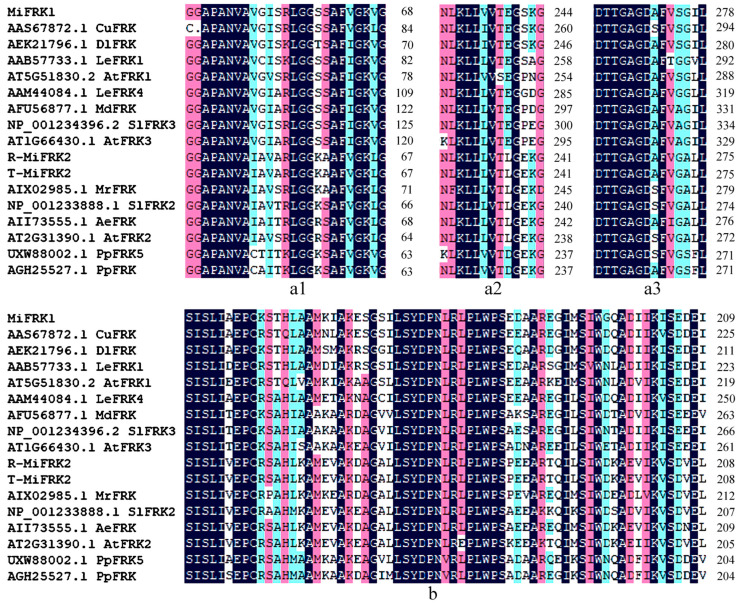
Conserved domain architecture of FRK proteins in mango and various other plant species. (**a1**–**a3**) Three signature structures of the pfkB family; (**b**) FRK-specific functional domain. CuFRK from *Citrus unshiu*, DlFRK from *Dimocarpus longan*, LeFRK1 from *Solanum lycopersicum*, AtFRK1 from *Arabidopsis thaliana*, LeFRK4 from *Solanum lycopersicum*, MdFRK from *Malus domestica*, SlFRK3 from *Solanum lycopersicum*, AtFRK3 from *Arabidopsis thaliana*, MrFRK from *Morella rubra*, SlFRK2 from *Solanum lycopersicum*, AeFRK from *Actinidia eriantha*, AtFRK2 from *Arabidopsis thaliana*, PpFRK5 from *Pyrus pyrifolia*, PpFRK from *Prunus persica*.

**Figure 2:**
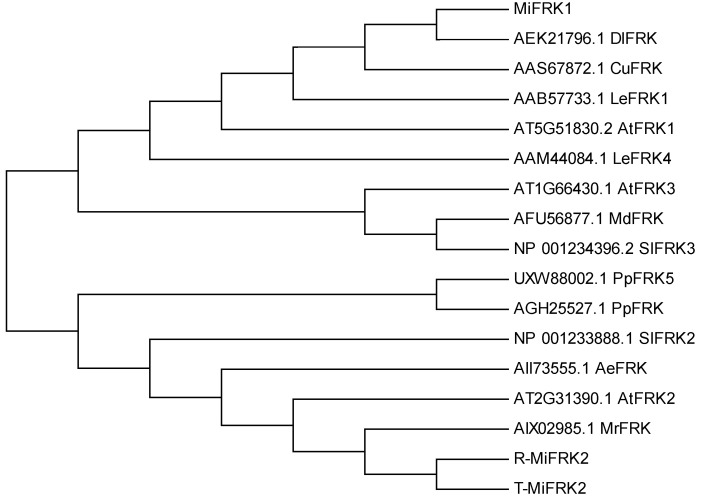
Phylogenetic analysis of FRK protein sequences across plant species. DlFRK from *Dimocarpus longan*, CuFRK from *Citrus unshiu*, LeFRK1 from *Solanum lycopersicum*, AtFRK1 from *Arabidopsis thaliana*, LeFRK4 from *Solanum lycopersicum*, AtFRK3 from *Arabidopsis thaliana*, MdFRK from *Malus domestica*, SlFRK3 from *Solanum lycopersicum*, PpFRK5 from *Pyrus pyrifolia*, PpFRK from *Prunus persica*, SlFRK2 from *Solanum lycopersicum*, AeFRK from *Actinidia eriantha*, AtFRK2 from *Arabidopsis thaliana*, MrFRK from *Morella rubra*.

Structural characterization demonstrated that both MiFRK1 and MiFRK2 proteins contain highly conserved functional domains, including the characteristic pfkB family signature sequence, FRK-specific sugar-binding domains, and ATP-binding motifs (Figure 1). Phylogenetic analysis revealed distinct evolutionary relationships among these proteins. MiFRK1 exhibited the closest homology with DlFRK (RefSeq: AEK21796.1) from longan (*Dimocarpus longan*) and CuFRK (RefSeq: AAS67872.1) from satsuma mandarin. In contrast, MiFRK2 clustered more closely with AtFRK2 (RefSeq: AT2G31390.1) from *Arabidopsis thaliana* and MrFRK protein (RefSeq: AIX02985.1) from Chinese bayberry (Figure 2).

### 2.2. Generation and Phenotypic Analysis of Transgenic Tomato

To functionally characterize mango *MiFRK1* and *MiFRK2* and examine the potential effects of intervarietal SNPs on gene function, we successfully transformed three FRK genes (*R-MiFRK2*, *T-MiFRK2,* and *MiFRK1*) into Micro-Tom tomato. PCR-based screening identified four independent *MiFRK1*-positive transgenic lines, six *T-MiFRK2*-positive lines, and eight *R-MiFRK2*-positive lines (Figure S4).

**Figure 3:**
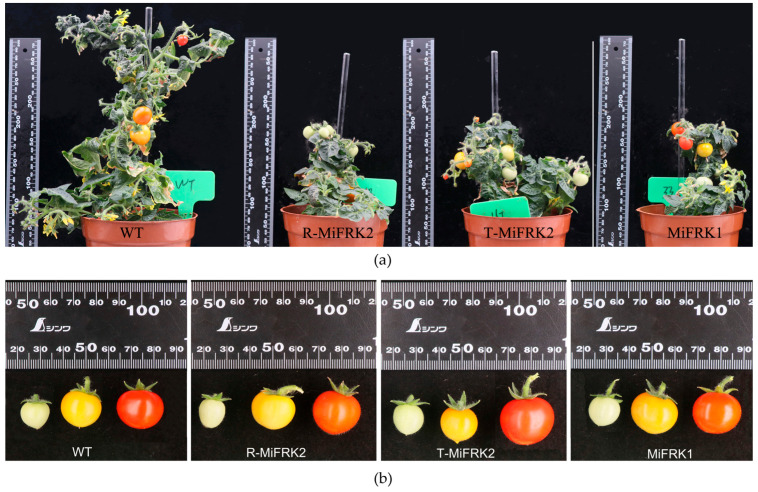
Phenotypic comparison between *MiFRK*-overexpressing and wild-type (WT) tomato plants. (**a**) Whole-plant phenotype. (**b**) Fruit phenotype.

Following the germination of resistant seedlings from the seeds of positive transgenic lines, the T_1_ plants were transplanted into growth containers for phenotypic analysis. The results demonstrated that all T_1_ transgenic tomato plants individually expressing the three mango *FRK* genes were significantly shorter than the controls, with no notable differences in fruit shape or size (Figure 3).

### 2.3. Enzymatic Activity Analysis in MiFRK-Overexpressing Tomato Fruits

**Figure 4:**
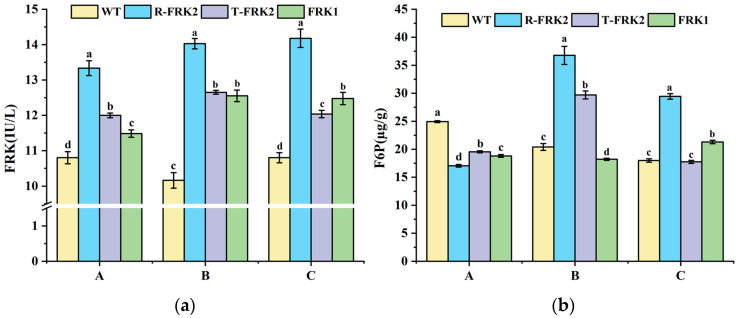
FRK activity and F6P content in transgenic tomato fruits across three developmental stages: young fruit (A), color-turning (B), and ripening (C). (**a**) FRK activity in transgenic tomato fruits across three developmental stages, (**b**) F6P content in transgenic tomato fruits across three developmental stages. Bars marked with different lowercase letters (a, b, c, d) indicate a significant difference at the 0.05 probability level.

Fruits from the T_1_ generation transgenic plants exhibited significantly higher FRK enzymatic activity across all developmental stages compared to WT (Figure 4a), demonstrating that both *MiFRK1* and *MiFRK2* isoforms encode functional enzymes capable of catalyzing fructose phosphorylation to F6P. Notably, *R-MiFRK2*-positive plants consistently exhibited the highest FRK activity across all three developmental phases, suggesting that the identified SNPs may enhance the catalytic efficiency of the encoded protein.

F6P, as the catalytic product of FRK, displayed a non-linear relationship with FRK enzyme activity across different developmental stages in the transgenic lines of three *MiFRKs* (Figure 4b): At the young fruit stage, all transgenic lines of three *MiFRK* genes exhibited significantly lower F6P levels than WT. At the color-turning stage, only *R-MiFRK2* and *T-MiFRK2* transgenic lines accumulated higher F6P contents compared to WT, while *MiFRK1* lines retained reduced F6P contents. Notably, at the ripening stage, an elevated F6P content was detected exclusively in *R-MiFRK2* and *MiFRK1* transgenic plants. These findings indicate that F6P undergoes dynamic developmental-stage-specific regulation.

### 2.4. Carbohydrate Content Analysis in MiFRK-Overexpressing Tomato Fruits

**Figure 5:**
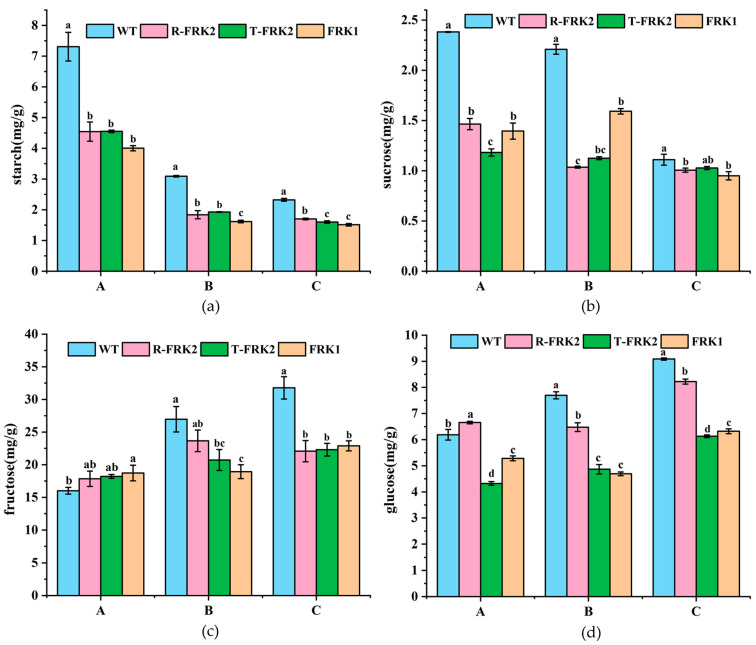
Major carbohydrate contents in transgenic tomato fruits across three developmental stages: young fruit (A), color-turning (B), and ripening (C). (**a**) Starch contents in transgenic tomato fruits across three developmental stages, (**b**) Sucrose contents in transgenic tomato fruits across three developmental stages, (**c**) Fructose contents in transgenic tomato fruits across three developmental stages, (**d**) Glucose contents in transgenic tomato fruits across three developmental stages. Bars marked with different lowercase letters (a, b, c, d) indicate a significant difference at the 0.05 probability level.

Metabolic analysis of major carbohydrate components (starch, sucrose, fructose, and glucose) in T_1_ generation transgenic tomato fruits expressing mango *FRK* genes revealed significant alterations in carbohydrate metabolism compared to WT plants (Figure 5). Starch content analysis showed a consistent developmental decline across all lines, with all three *MiFRK*-overexpressing transgenic lines exhibiting significantly lower starch accumulation than WT at each developmental stage (*p* < 0.05), suggesting FRK-mediated acceleration of starch catabolism.

Sucrose accumulation patterns differed markedly between genotypes. While WT tomatoes displayed the characteristic progressive decline in sucrose content during development, the three *FRK* transgenic lines showed distinct accumulation patterns (Figure 5b). Quantitative analysis demonstrated that *FRK*-overexpressing lines maintained significantly lower sucrose levels than WT controls throughout development (*p* < 0.05), except for *T-MiFRK* lines at the ripening stage. These results indicate that mango *MiFRK1* and *MiFRK2* overexpression significantly inhibits sucrose accumulation in tomato fruits.

The hexose (fructose and glucose) accumulation profiles revealed developmental stage-specific regulation (Figure 5c,d). At the young fruit stage, *MiFRK1*-overexpressing lines accumulated significantly higher fructose contents while *R-MiFRK2*-overexpressing lines showed elevated glucose levels compared to WT controls (*p* < 0.05). However, during both color-turning and ripening stages, all FRK-overexpressing fruits exhibited significantly reduced accumulation of both sugars relative to WT (*p* < 0.05), demonstrating substantial suppression of hexose accumulation during late fruit development.

### 2.5. Sugar Metabolism-Related Parameter Analysis in FRK-Overexpressing Tomato Fruits

**Figure 6:**
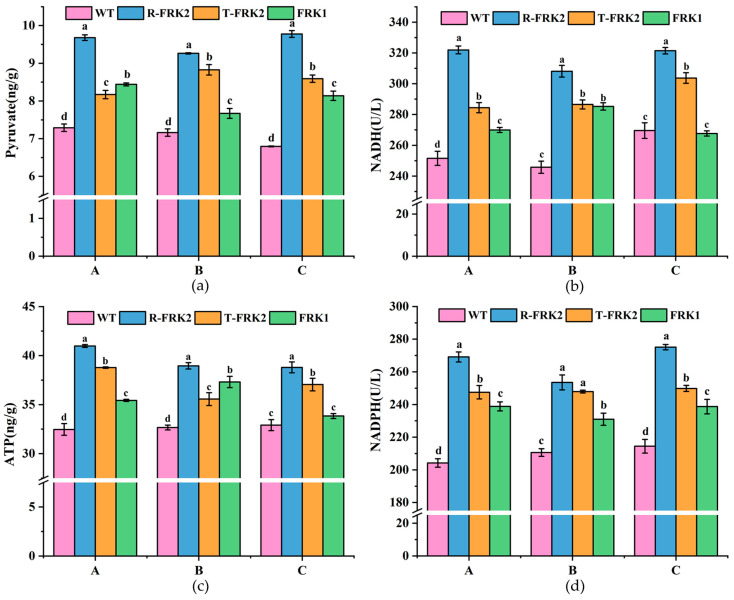
Analysis of sugar metabolism-related indicators in transgenic tomato fruits across three developmental stages: young fruit (A), color-turning (B), and ripening (C). (**a**) Analysis of pyruvate in transgenic tomato fruits across three developmental stages, (**b**) Analysis of NADH in transgenic tomato fruits across three developmental stages, (**c**) Analysis of ATP in transgenic tomato fruits across three developmental stages, (**d**) Analysis of NADPH in transgenic tomato fruits across three developmental stages. Bars marked with different lowercase letters (a, b, c, d) indicate a significant difference at the 0.05 probability level.

Metabolic profiling of key glycolytic intermediates revealed significant alterations in pyruvate, NADH, and ATP levels during fruit development in *MiFRK*-overexpressing lines (Figure 6). Comparative analysis with WT controls demonstrated that T_1_ transgenic fruits accumulated substantially higher pyruvate contents (*p* < 0.05), with *R-MiFRK1* lines exhibiting the most pronounced elevation across all developmental stages. Quantitative measurements showed concurrent increases in both NADH activity and ATP content in transgenic lines (*p* < 0.05), except in *MiFRK1* lines at the ripening stage, which showed no significant difference relative to controls. Notably, *R-MiFRK2* lines maintained the highest NADH activity and ATP content throughout fruit development.

Analysis of NADPH (Figure 6d), a critical metabolic indicator of pentose phosphate pathway activity, showed significantly enhanced levels in all *MiFRK* transgenic lines relative to WT controls (*p* < 0.05). Among the transgenic variants, *R-MiFRK2* lines exhibited the most substantial NADPH levels, with both *R-MiFRK2* and *T-MiFRK2* lines demonstrating significantly greater activity than *MiFRK1* lines (*p* < 0.05). These findings collectively indicate that *FRK* overexpression enhances glycolytic flux and promotes sugar metabolism conversion in tomato fruits. The consistent metabolic advantage observed in *R-MiFRK2* lines suggests that this particular variant may have a superior metabolic activation capacity compared to the other transgenic lines.

### 2.6. Correlation Analysis of Physiological Parameters in MiFRK-Overexpressing Tomato Fruits

To systematically evaluate the metabolic network regulated by *MiFRK* genes, we performed Pearson correlation analysis of key physiological parameters in T_1_ transgenic tomato fruits (Table 1). The analysis revealed that FRK enzyme activity showed highly significant positive correlations with multiple glycolytic intermediates and energy metabolites, including pyruvate (*p* < 0.01), NADPH (*p* < 0.01), NADH (*p* < 0.01), ATP (*p* < 0.01), and F6P (*p* < 0.01). In contrast, inverse correlations were observed between FRK activity and both fructose (*p* < 0.01) and starch (*p* < 0.05) contents. Notably, starch content showed highly significant positive correlations (all *p* < 0.01) with sucrose, glucose, and fructose levels. Additionally, F6P content showed significant positive associations with pyruvate (*p* < 0.01), NADH (*p* < 0.05), ATP (*p* < 0.05), and NADPH (*p* < 0.01).

These results collectively demonstrate that *MiFRK* overexpression preferentially activates sugar catabolic pathways while simultaneously inhibiting sugar accumulation, indicating a metabolic shift from carbohydrate storage toward energy production and biosynthetic precursor generation.

### 2.7. Transcriptome Analysis of T_1_ Generation Transgenic Tomato Fruits

To investigate the molecular mechanisms underlying the metabolic alterations induced by *MiFRK* overexpression, we performed comparative transcriptomic profiling of transgenic and WT tomato fruits.

**Figure 7:**
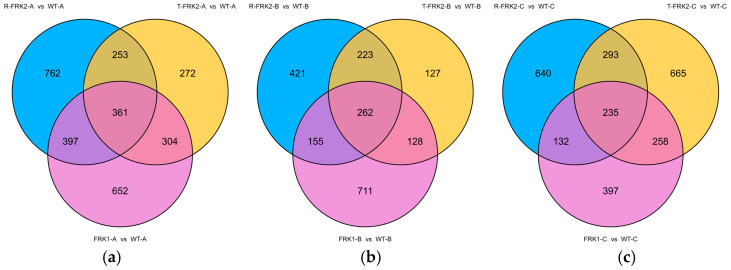
Venn diagram of DEGs in transgenic tomato plants of three different stage: young fruit stage (**a**), color-turning stage (**b**), and ripening stage (**c**).

In the young fruit stage, our comparative transcriptomic analysis identified 361 consistently differentially expressed genes (DEGs) shared across all three *MiFRK*-overexpressing T_1_ transgenic lines compared to WT controls (Figure 7a). Notably, within the glycolysis/gluconeogenesis pathway (ko00010), *6-phosphofructokinase* (Solyc05g024230.2)—a key regulatory enzyme catalyzing the irreversible conversion of F6P to fructose-1,6-bisphosphate—was significantly upregulated, indicating enhanced glycolytic flux at this major regulatory checkpoint. Additionally, two *alcohol dehydrogenase* genes (Solyc09g015070.3 and Solyc04g064710.3), which mediate the interconversion of acetaldehyde and ethanol, exhibited coordinated upregulation. Beyond glycolysis, we observed upregulation of *succinate dehydrogenase* (Solyc04g055020.2, Solyc04g055030.2) from the TCA cycle (ko00020) and *transketolase* (Solyc01g018020.2) from the pentose phosphate pathway.

During the color-turning stage, transcriptomic comparison between T_1_ transgenic plants (harboring three *FRK* genes) and WT plants revealed 262 common DEGs in fruit tissues (Figure 7b). Within the glycolysis/gluconeogenesis pathway (ko00010), key enzymes—including *pyruvate kinase* (Solyc06g051930.3), *acetate/butyrate-CoA ligase* (Solyc02g082910.3), and *phosphoenolpyruvate carboxylase* (Solyc04g076880.3)—were significantly downregulated. Conversely, in the pentose and glucuronate interconversion pathway, two *pectinesterase* isoforms (Solyc08g079235.1 and novel.141) showed consistent upregulation.

In ripening-stage fruits, transcriptomic analysis of T_1_ FRK transgenic plants identified 235 common DEGs relative to WT (Figure 7c). Within the pentose and glucuronate interconversion pathway, key cell wall-modifying enzymes—*polygalacturonase* (Solyc05g005170.3) and *pectinesterase* (Solyc11g019910.1)—were significantly upregulated. In the starch and sucrose metabolism pathway (ko00500), *endoglucanase* (Solyc08g083210.3) was upregulated, which could facilitate cellulose breakdown into cellodextrin and cellobiose, whereas *glucan endo-1,3-β-glucosidase 4* (Solyc01g059965.1, Solyc01g060020.3) was downregulated, potentially inhibiting the conversion of 1,3-β-glucan to D-glucose. Furthermore, in the Calvin cycle carbon fixation pathway, two critical enzymes—*phosphoribulokinase* (Solyc08g076220.3) and *glyceraldehyde-3-phosphate dehydrogenase* (Solyc04g009030.3)—were upregulated, suggesting enhanced carbon assimilation.

**Figure 8:**
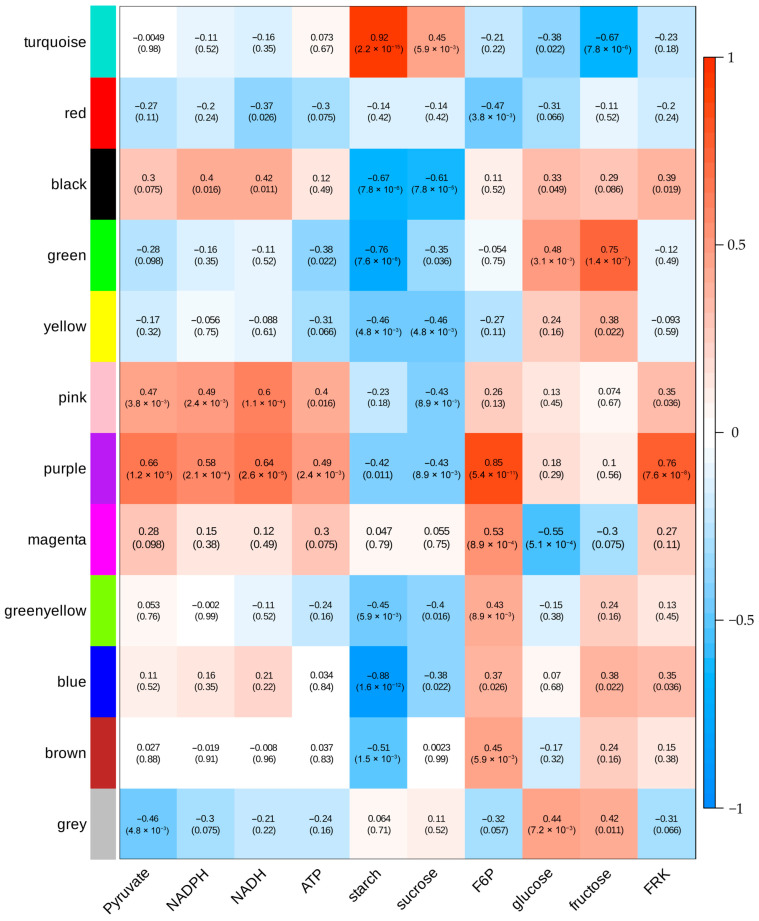
Analysis of the correlation between different gene modules and physiological indicators.

To identify key regulatory genes involved in sugar metabolism-related physiological traits, we conducted WGCNA, which grouped the DEGs into 12 distinct modules (Figure 8). Among these, the turquoise module exhibited a strong positive correlation with starch and sucrose accumulation but significant negative correlations with fructose levels. Within this module, we identified 21 glycolysis/gluconeogenesis-associated genes, including downregulated *aldehyde dehydrogenase (NAD+)* (Solyc06g060250.3) and *acetate/butyrate-CoA ligase* (Solyc08g075800.2), along with upregulated *enolase* (*ENO*, Solyc02g063255.1) in *FRK* transgenic T_1_ fruits (Table S2). Additionally, the turquoise module contained 33 starch/sucrose metabolism genes comprising seven *endoglucanases* (Solyc08g082250.3 et al.), nine *glucan endo-1,3-β-glucosidases* (Solyc07g056310.3 et al.), three *β-glucosidases* (Solyc01g074040.2 et al.), three *trehalose-6-phosphate phosphatases* (Solyc03g007290.3 et al.), and three *trehalose-6-phosphate synthase/phosphatases* (Solyc02g071590.2 et al.) (Table S2). In contrast, the blue module exhibited a strong negative correlation with starch content and contained two key starch and sucrose metabolism pathway genes: *starch synthase* (Solyc03g083095.1) and *β-amylase* (Solyc07g052695.1).

The green module also showed significant negative correlation with starch accumulation, but positive correlations with glucose and fructose levels. This module contained two genes from the glycolysis/gluconeogenesis pathway including *phosphoenolpyruvate carboxylase* (Solyc04g076880.3), which catalyzes the conversion of phosphoenolpyruvate to oxaloacetate, and *6-phosphofructokinase 1* (Solyc07g045160.3), a rate-limiting enzyme in glycolysis. Additionally, we identified two starch metabolism-related genes, *glucan endo-1,3-β-glucosidase 4* (Solyc01g060020.3), which are involved in β-glucan degradation, and *β-amylase* (*β-AMY*, Solyc07g052690.3), which catalyzes starch breakdown to maltose, that displayed significantly higher expression levels in young fruits of the overexpression lines compared to controls.

The purple module was highly significantly positively correlated with pyruvate, NADPH, NADH, ATP, F6P, and FRK, but highly significantly negatively correlated with starch and sucrose. Notably, it contained *β-fructofuranosidase* (Solyc03g083910.3), which irreversibly catalyzes the breakdown of sucrose into glucose and fructose. potentially explaining its inverse relationship with sucrose.

The magenta module also showed a positive correlation with F6P but negative correlation with glucose. This module included *glucan endo-1,3-β-glucosidase* (Solyc12g014420.2) from the starch and sucrose metabolism pathway, which liberates glucose from 1,3-β-glucan, and *malate dehydrogenase* (Solyc08g066360.3), linking pyruvate metabolism to the TCA cycle.

### 2.8. Validation of Key Genes by qRT-PCR

To validate the relative expression patterns of key genes identified through transcriptome analysis, two critical genes involved in sugar metabolism (*ENO* and *SP*) were selected for qRT-PCR analysis. The expression patterns were examined in fruits of both wild-type and MiFRK-overexpressing tomato lines across different developmental stages. As shown in Supplementary Figure S5, the relative expression levels of all selected genes demonstrated high consistency with the FPKM values, with a coefficient of determination (R^2^) of 0.938 in the linear regression analysis. These results confirm the reliability of the transcriptome data and subsequent interpretations.

## 3. Discussion

*FRKs* play pivotal regulatory roles in carbon partitioning, developmental processes, and abiotic stress responses [28]. In apple, the expression dynamics of *FRK* isoforms exhibit distinct temporal patterns during fruit development: while *MdFK2* shows peak expression in young fruits followed by a sharp decline in later developmental stages, *MdFK1* demonstrates progressive upregulation as fruits approach maturity [29]. In our preliminary studies, we observed distinct expression patterns of mango *MiFRK1* and *MiFRK2* during fruit development. Notably, multiple SNP variations were identified in *MiFRK2* between high-sugar and low-sugar mango cultivars. To elucidate the functions of mango *MiFRK1* and *MiFRK2* and investigate whether the intervarietal SNPs affect gene functionality, we conducted genetic transformations of tomato with *MiFRK1* and two allelic variants of *MiFRK2* (*R-MiFRK2* and *T-MiFRK2*) from the respective cultivars. Transgenic analysis demonstrated that all three *FRK* transgenes significantly enhanced FRK activity in T_1_ generation plants throughout fruit development. Quantitative assessment revealed that the *R-MiFRK2* overexpression lines consistently exhibited the highest enzymatic activity across all developmental timepoints, confirming that both *FRK* isoforms possess functional fructokinase activity capable of phosphorylating fructose to F6P. Notably, the observed activity difference between *R-MiFRK2* and *T-MiFRK2* suggests that the identified SNPs may confer functional modifications to the encoded proteins, with the *MiFRK2* allele from the low-sugar cultivar (‘Renong NO. 1’) unexpectedly displaying superior catalytic efficiency. Notably, although FRK activity was significantly elevated, the F6P accumulation profile showed inconsistent patterns. In particular, F6P levels in transgenic tomato fruits at early developmental stages were significantly lower than those in wild-type controls. This phenomenon may be attributed to either (1) substrate limitation caused by accelerated fructose phosphorylation, or (2) enhanced F6P utilization through FRK-mediated regulation of downstream metabolic pathways.

Analysis of starch, sucrose, fructose, and glucose levels revealed that *MiFRK* overexpression significantly reduced starch and sucrose accumulation in transgenic plants across all fruit developmental stages, while fructose and glucose levels were notably decreased only during mid-to-late development. Consistent with these findings, functional characterization of pear *FRK* (*PbFRK1*) demonstrated its ability to enhance hexokinase activity and reduce the sugar content in transgenic tomato plants [19]. Interestingly, no significant alteration in fructose content was observed in young tomato fruits overexpressing the *MiFRK* transgene. This finding implies that the marked decrease in F6P levels likely stems from *MiFRK*-induced activation of downstream glycolytic pathways, consequently enhancing F6P metabolic flux and turnover. These findings further demonstrate that mango *FRK* exerts different regulatory effects on tomato fruit metabolism across distinct developmental stages. This stage-specific phenomenon aligns with previous observations in tomato research, where *LeFRK2* was shown to be dispensable for starch biosynthesis during fruit development [30].

Key glycolytic metabolites (pyruvate, ATP, NADH) and PPP-derived NADPH were quantified in *MiFRK*-overexpressing tomato fruits at various developmental stages. Compared to wild-type controls, *FRK*-overexpressing lines consistently showed significantly higher pyruvate and NADPH accumulation compared to wild-type controls at all developmental stages. Notably, while *FRK1*-overexpressing fruits exhibited comparable ATP levels and NADH activity to wild-type controls specifically during ripening, all other transgenic lines showed significant increases in both parameters across all developmental phases. Among these, the *R-MiFRK2* line displayed the highest values for all analyzed metabolites throughout fruit development. These findings provide compelling evidence that heterologous expression of *MiFRK* in tomato actively promotes carbon partitioning through both the glycolytic and pentose phosphate pathways. Correlation analysis further supported these findings. FRK activity exhibited significant negative correlations with fructose (*p* < 0.01) and starch (*p* < 0.05) contents. Conversely, FRK enzyme activity showed highly significant positive correlations with pyruvate (*p* < 0.01), NADPH (*p* < 0.01), NADH (*p* < 0.01), ATP (*p* < 0.01), and F6P (*p* < 0.01). These findings suggest a potential regulatory role of FRK in carbon partitioning and energy metabolism, and thus it could serve as a target gene for the manipulation of these processes using gene-editing technologies. Interestingly, our findings appear to contrast with previous reports in other plant systems. For instance, Zhang et al. [31] observed that overexpression of *OsFRK3* in rice (*Oryza sativa*) seeds led to suppressed fructose accumulation while promoting starch biosynthesis. Similarly, Yang et al. [17] reported that elevated *MdFRK2* activity in apple leaves resulted in enhanced sorbitol metabolism and reduced sucrose metabolism, ultimately favoring starch accumulation. These opposing effects highlight the potential system-specific or isoform-dependent regulatory mechanisms of FRK in different plant tissues and species.

**Figure 9:**
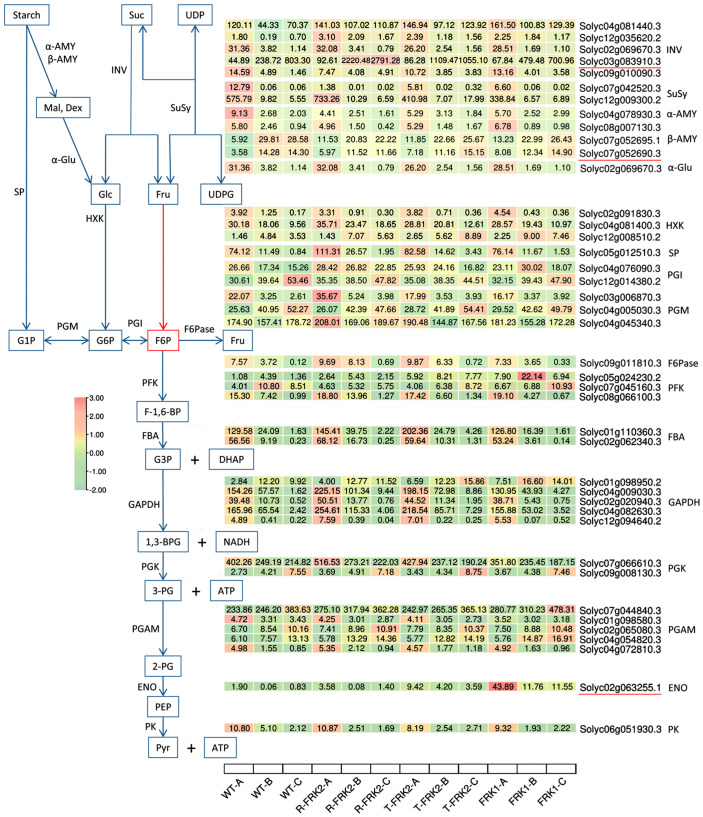
Heatmap of the expression of some genes related to sugar metabolism. Suc, Sucrose; UDP, Uridine diphosphate; Mal, Maltose; Dex, Dextrin; Glc, Glucose; Fru, Fructose; UDPG, UDP-glucose; G1P, Glucose-1-phosphate; G6P, Glucose-6-phosphate; F6P, Fructose-6-phosphate; F-1,6-BP, Fructose-1,6-bisphosphate; G3P, Glyceraldehyde-3-phosphate; DHAP, Dihydroxyacetone phosphate; 1,3-BPG, 1,3-Bisphosphoglycerate; NADH, Nicotinamide adenine dinucleotide (reduced form); 3-PG, 3-Phosphoglycerate; 2-PG, 2-Phosphoglycerate; PEP, Phosphoenolpyruvate; Pyr, Pyruvate; *α-AMY, α-Amylase; β-AMY, β-Amylase; INV, Invertase; SuSy, Sucrose synthase; α-Glu, α-Glucosidase; SP, Sucrose phosphorylase; HXK, Hexokinase; PGM, Phosphoglucomutase; PGI, Phosphoglucose isomerase; F6Pase, Fructose-6-phosphatase; PFK, Phosphofructokinase; FBA, Fructose-bisphosphate aldolase; GAPDH, Glyceraldehyde-3-phosphate dehydrogenase; PGK, Phosphoglycerate kinase; PGAM, Phosphoglycerate mutase; ENO, Enolase; PK, Pyruvate kinase*.

Based on transcriptomic and WGCNA analyses, we identified a set of key genes involved in the fructose kinase overexpression-mediated regulation of sugar metabolism (Figure 9). These include *β-fructofuranosidase* (Solyc03g083910.3), which catalyzes the irreversible hydrolysis of sucrose into glucose and fructose, *β-amylase* (Solyc07g052690.3), which exhibited a significant negative correlation with starch accumulation but positive correlations with glucose and fructose levels and mediates starch degradation to maltose, and *ENO* (Solyc02g063255.1), a key glycolytic enzyme that catalyzes the conversion of 2-phosphoglycerate to phosphoenolpyruvate (PEP), thereby directly facilitating pyruvate synthesis and enhancing energy metabolism efficiency.

*β-Fructofuranosidase* (*invertase*, Solyc03g083910.3) exhibited significant upregulation across all fruit developmental stages in *T-MiFRK2* and *R-MiFRK2* overexpression lines, whereas its expression was only markedly elevated at the young fruit and color-turning stages in *MiFRK1*-overexpressing lines. Notably, *alkaline/neutral invertase* (Solyc04g081440.3), which shares similar enzymatic activity, showed significantly increased expression throughout all developmental phases in all overexpression lines. These findings suggest that both enzymes coordinately participate in sucrose degradation in transgenic fruits. This observation aligns with previous studies in date palm (*Phoenix dactylifera*) [32] and rice [33], further confirming the pivotal role of invertases in plant sucrose metabolism. Regarding starch metabolism, *β-amylase* (Solyc07g052690.3) and its homologous gene (Solyc07g052695.1) displayed significantly higher expression levels in young fruits of overexpression lines compared to controls. Concurrently, *starch phosphorylase* (*SP*, Solyc05g012510.3) was also markedly upregulated in most transgenic fruit samples. These results indicate that *β-amylase* and *SP* collectively contribute to starch degradation in overexpression lines. This discovery is consistent with the functional evolution of the *β-amylase* gene family reported by Thalmann et al. [34] and the established role of *SP* in starch metabolism [35]. Of particular interest, *ENO* (Solyc02g063255.1) showed significantly enhanced expression in all transgenic lines, with a more than 10-fold upregulation observed at three developmental stages in *MiFRK1*-overexpressing lines. This dramatic increase likely promotes glycolytic flux and pyruvate accumulation. Correspondingly, the key glycolytic gene *GAPDH* was significantly upregulated in *R-MiFRK1* and *T-MiFRK1* overexpression lines. Previous studies in tobacco (*Nicotiana tabacum*) [36] and *Arabidopsis* [37] have demonstrated that both *enolase* and *GAPDH* participate in pyruvate synthesis and energy metabolism regulation, which corroborates our findings. These results provide novel insights into the regulatory mechanisms of sugar metabolism in fruits mediated by *FRK* gene overexpression.

## 4. Materials and Methods

### 4.1. Materials

#### 4.1.1. Plant Samples

Micro-Tom tomato seeds were obtained from Beijing Huayueyang Biotechnology (Beijing, China). Fruits from transgenic positive T_1_ generation plants and wild-type (WT) plants were harvested at three developmental stages: young fruit stage (A), color-turning stage (B), and ripening stage (C). Tissue samples from the positive transgenic lines were pooled for downstream analysis. All samples were immediately frozen in liquid nitrogen, ground into a fine powder, and stored at −80 °C until further use.

#### 4.1.2. Reagents

The Ezup Column Plant Genomic DNA Extraction Kit and Plant Total RNA Rapid Extraction Kit were purchased from Sangon Biotech (Shanghai) Co., Ltd. (Shanghai, China). The RevertAid First Strand cDNA Synthesis Kit was obtained from Thermo Fisher Scientific (Waltham, MA, USA). Trans Taq^®^-T DNA Polymerase and the pEASY^®^-Blunt Cloning Kit were provided by TransGen Biotech Co., Ltd. (Beijing, China). The DM 2000 DNA Marker, pM-DTM19-T Vector Cloning Kit, *E. coli* DH5α Competent Cells, FastDigest restriction enzymes, and T4 DNA Ligase were purchased from TaKaRa Bio (Beijing) Co., Ltd. (Beijing, China). The Universal DNA Purification and Recovery Kit was acquired from TIANGEN Biotech (Beijing) Co., Ltd. (Beijing, China). All other reagents were of analytical grade (AR) and domestically sourced.

Primers were designed using Primer Premier 5.0 and synthesized by BGI TECH SOLUTIONS (BEIJING LIUHE) Co., Ltd. (Beijing, China). The relevant primer sequences are listed in Table S1.

### 4.2. Methods

#### 4.2.1. Analysis of MiFRK1 and MiFRK2 Protein in Two Varieties

cDNA and Protein sequence alignment was performed using DNAMAN 8 software and the CLUSTALW online tool (https://www.genome.jp/tools-bin/clustalw (accessed on 16 June 2024)). A Neighbor-Joining phylogenetic tree was constructed using MEGA 7 software with 1000 bootstrap replications.

#### 4.2.2. Tomato Genetic Transformation

The target genes were ligated downstream of the CaMV 35S promoter in the plant expression vector pCAMBIA2300 using a double enzyme digestion method. The recombinant vectors were then introduced into *Agrobacterium tumefaciens* strain GV3101 using the liquid nitrogen freeze–thaw method. Cotyledon explants of Micro-Tom tomato were co-cultivated with the transformed *Agrobacterium* (on MS medium supplemented with 2.0 mg/L ZT and 0.2 mg/L IAA). Selection was performed on shoot induction medium (MS + 2.0 mg/L ZT + 0.2 mg/L IAA) containing 50 mg/L kanamycin (Kan) and 250 mg/L cefotaxime (Cef). Putative transgenic shoots regenerated on the selection medium were transferred to a rooting medium (MS + 0.1 mg/L IAA + 50 mg/L Kan + 250 mg/L Cef). Rooted plantlets were acclimatized in soil and grown in greenhouse conditions. Putative transgenic lines were verified by PCR, with primers listed in Table S1.

#### 4.2.3. Determination of Primary Indicator of Sugar Metabolism in Tomato Fruits

The physiological indicators were measured using commercial assay kits following the manufacturer’s protocols. Specifically, starch content was determined using the Starch Content Assay Kit (ADS-F-DF001), sucrose content was analyzed using the Sucrose Content Assay Kit (Anthrone Colorimetric Method, ADS-F-DF016), F6P levels were assessed using the F6P Content Assay Kit (ADS-F-T018), glucose content was measured using the Glucose Content Assay Kit (GOPOD Oxidase Method, ADS-F-TDX002), and fructose content was quantified using the Fructose Content Assay Kit (Resorcinol Method, ADS-F-TDX041), all of which were obtained from Aidisheng Biological Technology Co., Ltd. (Yancheng, China). Additionally, the following ELISA kits were purchased from Liborui Biotechnology Co., Ltd. (Wuhan, China): Plant Pyruvate Enzyme-Linked Immunosorbent Assay (ELISA) Kit, Plant Reduced Nicotinamide Adenine Dinucleotide (NADH) ELISA Kit, Plant Reduced Nicotinamide Adenine Dinucleotide Phosphate (NADPH) ELISA Kit, and Plant Adenosine Triphosphate (ATP) ELISA Kit.

#### 4.2.4. Transcriptome Analysis of Tomato Fruits

Library preparation and sequencing were performed by MetWare Biotechnology Co., Ltd. (Wuhan, China). Total RNA was extracted from the samples using a combination of CTAB-PBIOZOL and ethanol precipitation. The RNA quality and quantity were assessed using a Qubit fluorometer and a Qsep400 bioanalyzer (BIOptic, Inc., Xinbei, China). Sequencing libraries were constructed following a strand-specific protocol. Briefly, mRNA was enriched with Oligo(dT) magnetic beads, fragmented, and reverse-transcribed into cDNA. Strand specificity was achieved by incorporating dUTP during the second-strand synthesis. The cDNA fragments then underwent end-repair, dA-tailing, and adapter ligation. After purification and size selection, the libraries were amplified by PCR. The final library quality was confirmed before being pooled and sequenced using the Illumina HiSeq 4500 platform (Illumina San Diego, CA, USA) to generate 150 bp paired-end reads.

Raw sequencing reads were processed with fastp to remove adapters and low-quality sequences, resulting in clean reads for subsequent analysis. The clean reads were then aligned to the reference genome using HISAT2. Transcript assembly and novel gene prediction were performed using StringTie v2.2.3. Gene expression levels were calculated as FPKM (Fragments Per Kilobase per Million mapped reads) values. DEGs were identified using DESeq2 with thresholds of |log_2_FC| ≥ 1 and FDR < 0.05 (Benjamini–Hochberg correction). Finally, functional enrichment analysis of Gene Ontology (GO) terms and Kyoto Encyclopedia of Genes and Genomes (KEGG) pathways was performed based on the hypergeometric test, and a weighted Gene Co-Expression Network Analysis (WGCNA) was performed using the MetWare Cloud Platform (https://cloud.metware.cn/ (accessed on 21 January 2025)) to identify trait-associated gene modules.

#### 4.2.5. Validation of Transcriptome Data by Quantitative Real-Time PCR (qRT-PCR)

qRT-PCR was performed to validate the transcriptome sequencing results. Briefly, total RNA was first reverse-transcribed into cDNA using the *TransScript*^®^ One-Step gDNA Removal and cDNA Synthesis SuperMix kit (TransGen Biotech Beijing, China). Subsequently, qRT-PCR assays were carried out on a Roche Lightcycler^®^ 480 instrument using the *PerfectStart*^®^ Green qPCR SuperMix (TransGen Biotech, China) following the manufacturer’s protocols. The actin gene (ACT) was used as an internal control, and the primer sequences for the target genes are listed in Table S1.

#### 4.2.6. Data Analysis

Statistical analysis and graphical visualization were performed using SPSS 26 (IBM Corp., Armonk, NY, USA) and Origin 2018 (OriginLab Corp., Northampton, MA, USA).

## 5. Conclusions

This study demonstrates that mango *MiFRK1* and *MiFRK2* (including *R-MiFRK2* and *T-MiFRK2* variants) function as active FRKs, catalyzing fructose phosphorylation to F6P while simultaneously promoting downstream F6P catabolism. Notably, R-MiFRK2 exhibits superior enzymatic activity and more robust metabolic regulation compared to other FRK isoforms. Transcriptomic and metabolic analyses reveal that overexpression of mango *MiFRKs* inhibit sugar content by altering the expression of key genes involved in sugar metabolism, including sucrose degradation genes (*invertases*), starch breakdown genes (*β-amylases*) and glycolytic genes (*enolase*). Notably, *MiFRK1* and *MiFRK2* exhibit differential regulatory effects on these metabolic pathways, suggesting functional divergence between the two isoforms. These findings provide new insights into the molecular mechanisms through which *FRKs* regulate sugar metabolism in mango fruit, highlighting their potential as targets for improving fruit quality through metabolic engineering.

## Figures and Tables

**Table 1:** Correlation analysis of physiological indicators of *MiFRK*-overexpressing tomato fruits.

	Pyruvate	NADPH	NADH	ATP	Starch	Sucrose	F6P	Glucose	Fructose	FRK
Pyruvate	1	0.989 **	0.861 **	0.904 **	−0.688 *	−0.495	0.786 **	−0.283	−0.830 **	0.944 **
NADPH	0.989 **	1	0.888 **	0.916 **	−0.648 *	−0.438	0.794 **	−0.234	−0.782 **	0.928 **
NADH	0.861 **	0.888 **	1	0.944 **	−0.353	−0.131	0.633 *	−0.016	−0.582 *	0.731 **
ATP	0.904 **	0.916 **	0.944 **	1	−0.464	−0.225	0.634 *	−0.158	−0.648 *	0.765 **
starch	−0.688 *	−0.648 *	−0.353	−0.464	1	0.783 **	−0.308	0.844 **	0.915 **	−0.622 *
sucrose	−0.495	−0.438	−0.131	−0.225	0.783 **	1	−0.349	0.614 *	0.810 **	−0.573
F6P	0.786 **	0.794 **	0.633 *	0.634 *	−0.308	−0.349	1	0.236	−0.455	0.913 **
glucose	−0.283	−0.234	−0.016	−0.158	0.844 **	0.614 *	0.236	1	0.688 *	−0.138
fructose	−0.830 **	−0.782 **	−0.582 *	−0.648 *	0.915 **	0.810 **	−0.455	0.688 *	1	−0.759 **
FRK	0.944 **	0.928 **	0.731 **	0.765 **	−0.622 *	−0.573	0.913 **	−0.138	−0.759 **	1

Statistical significance indicators: ** *p* < 0.01 (highly significant), * *p* < 0.05 (significant).

## Data Availability

All relevant data is available within the manuscript and Supplementary Materials.

## Supplementary Materials

The following supporting information can be downloaded at: https://www.mdpi.com/article/10.3390/plants14223526/s1, Table S1, Name, sequence, and application of the primers employed in this study; Table S2, Key genes involved in sugar metabolism across different modules; Figure S1, Comparison of *FRK1* cDNA sequences between ‘Renong No. 1’ and ‘Tainong No. 1’; Figure S2, Comparison of *FRK2* cDNA sequences between ‘Renong No. 1’ and ‘Tainong No. 1’; Figure S3, Comparison of *FRK1* and *FRK2* protein sequences between ‘Renong No. 1’ and ‘Tainong No. 1; Figure S4, PCR validation of resistant strain lines; Figure S5, Linear correlation between relative expression level and FPKM value.

## References

[1] Chen T., Qin G., Tian S. (2020). Regulatory network of fruit ripening: Current understanding and future challenges. New Phytol..

[2] Ruan Y.L. (2014). Sucrose metabolism: Gateway to diverse carbon use and sugar signaling. Annu. Rev. Plant Biol..

[3] Shangguan L., Song C., Leng X., Kayesh E., Sun X., Fang J. (2014). Mining and comparison of the genes encoding the key enzymes involved in sugar biosynthesis in apple, grape, and sweet orange. Sci. Hortic..

[4] Sadka A., Shlizerman L., Kamara I., Blumwald E. (2019). Primary metabolism in citrus fruit as affected by its unique structure. Front. Plant Sci..

[5] Granot D., Kelly G., Stein O., David-Schwartz R. (2014). Substantial roles of hexokinase and fructokinase in the effects of sugars on plant physiology and development. J. Exp. Bot..

[6] Granot D. (2007). Role of tomato hexose kinase. Funct. Plant Biol..

[7] Ofer S., David G. (2018). Plant fructokinases: Evolutionary, developmental, and metabolic aspects in sink tissues. Front. Plant Sci..

[8] German M.A., Dai N., Matsevitz T., Hanael R., Petreikov M., Bernstein N., Ioffe M., Shahak Y., Schaffer A., Granot D. (2003). Suppression of fructokinase encoded by LeFRK2 in tomato stem inhibits growth and causes wilting of young leaves. Plant J..

[9] Qin Q.P., Zhang S.L., Chen J.W., Xie Y.F., Chen K.S., Syed A. (2004). Isolation and expression analysis of fructokinase genes from Citrus. Acta Bot. Sin..

[10] Suzuki Y., Odanaka S., Kanayama Y. (2001). Fructose content and fructose-related enzyme activity during the fruit development of apple and Japanese pear. J. Jpn. Soc. Hortic. Sci..

[11] Qin Q.P., Cui Y.Y., Zhang L.L., Lin F.F., Lai Q.X. (2014). Isolation and induced expression of a fructokinase gene from loquat. Russ. J. Plant Physiol..

[12] Chen X., Shi L.Y., Shao J.R., Chen W., Zheng Y.H., Yang Z.F. (2016). Molecular cloning and expression analysis of MrFRK2 in Chinese bayberry during fruit ripening. Acta Hortic. Sin..

[13] Zhao J.H., Yin Y., Li H.X., Wang Y.J., Li Y.L., An W. (2018). Cloning and expression analysis of fructokinase gene (LbFRK7) from wolfberry (Lycium barbarum Linn.). Acta Bot. Boreali-Occident. Sin..

[14] Luo D.L., Ba L.J., Wang H.L., Zhang Q.M. (2025). Cloning, expression and enzymatic activity analysis of the fructokinase gene HpFRK2 in red pitaya (*Hylocereus polyrhizus*). Chin. J. Trop. Crops.

[15] Wang Q.F., Gong S.S., Bai Y., Liu N., Wang P.Y., Wu T. (2022). Changes in fructose, fructokinase and its gene in Morinda citrifolia during fruit development. J. Trop. Subtrop. Bot..

[16] Lü W.Y., Zhang L.Q., Gao Q.H., Duan K. (2020). Cloning and expression analysis of fructokinase gene FaFRK3 from ‘Benihoppe’ strawberry. Acta Agric. Shanghai.

[17] Yang J.J., Zhu L.C., Cui W.F., Zhang C., Li D.X., Ma B.Q., Cheng L.L., Ruan Y.L., Ma F.W., Li M.J. (2018). Increased activity of MdFRK2, a high-affinity fructokinase, leads to upregulation of sorbitol metabolism and downregulation of sucrose metabolism in apple leaves. Hortic. Res..

[18] Su J., Zhang C.X., Zhu L.C., Yang N.X., Yang J.J., Ma B.Q., Ma F.W., Li M.J. (2021). MdFRK2-mediated sugar metabolism accelerates cellulose accumulation in apple and poplar. Biotechnol. Biofuels.

[19] Zhao B., Yi X., Liu S., Qi K., Zhang S., Wu X. (2024). The PbFRK1 gene from pear fruit affects sugar accumulation. Sci. Hortic..

[20] Tao X., Zhu R.X., Gong X., Wu L., Zhang S.L., Zhao J.R., Zhang H.P. (2022). Fructokinase gene PpyFRK5 plays an important role in sucrose accumulation of pear fruit. Acta Hortic. Sin..

[21] Odanaka S., Bennett A.B., Kanayama Y. (2002). Distinct physiological roles of fructokinase isozymes revealed by gene-specific suppression of Frk1 and Frk2 expression in tomato. Plant Physiol..

[22] Damari-Weissler H., Rachamilevitch S., Aloni R., German M.A., Cohen S., Zwieniecki M.A., Holbrook N.M., Granot D. (2009). LeFRK2 is required for phloem and xylem differentiation and the transport of both sugar and water. Planta.

[23] Ma B., Yuan Y., Gao M., Qi T., Li M., Ma F. (2018). Genome-wide identification, molecular evolution, and expression divergence of aluminum-activated malate transporters in apples. Int. J. Mol. Sci..

[24] Yang J.J., Zhan R.L., Wang L., Li J.Q., Ma B.Q., Ma F.W., Li M.J. (2023). Overexpression of MdFRK2 enhances apple drought resistance by promoting carbohydrate metabolism and root growth under drought stress. Hortic. Plant J..

[25] Fuchs Y., Pesis E., Zauberman G. (1980). Changes in amylase activity, starch and sugar contents in mango fruit pulp. Sci. Hortic..

[26] Li L., Wu H.X., Ma X.W., Xu W.T., Liang Q.Z., Zhan R.L., Wang S.B. (2020). Transcriptional mechanism of differential sugar accumulation in pulp of two contrasting mango (*Mangifera indica* L.) cultivars. Genomics.

[27] Zheng B., Wang S.B., Li X.Y., Wu H.X., Ma X.W., Liang Q.Z., Xu W.T., Li L. (2022). Cloning, expression and bioinformatic analysis of MiFRK genes in mango varieties with high or low sugar content. J. Fruit Sci..

[28] Zörb C., Schmitt S., Mühling K.H. (2010). Proteomic changes in maize roots after short-term adjustment to saline growth conditions. Proteomics.

[29] Li M., Feng F., Cheng L. (2012). Expression patterns of genes involved in sugar metabolism and accumulation during apple fruit development. PLoS ONE.

[30] Dai N., German M.A., Matsevitz T., Hanael R., Swartzberg D., Yeselson Y., Petreikov M., Schaffer A.A., Granot D. (2002). LeFRK2, the gene encoding the major fructokinase in tomato fruits, is not required for starch biosynthesis in developing fruits. Plant Sci..

[31] Zhang Z.F., Tan J.X., Chen Y.T., Sun Z.Y.Q., Yan X., Ouyang J.X., Wang X. (2023). New fructokinase, OsFRK3, regulates starch accumulation and grain filling in rice. J. Agric. Food Chem..

[32] Malek J.A., Mathew S., Mathew L.S., Younuskunju S., Mohamoud Y.A., Suhre K. (2020). Deletion of beta-fructofuranosidase (invertase) genes is associated with sucrose content in Date Palm fruit. Plant Direct.

[33] Wang Z.Z., Li H., Weng Y.X. (2024). A neutral invertase controls cell division besides hydrolysis of sucrose for nutrition during germination and seed setting in rice. iScience.

[34] Thalmann M., Coiro M., Meier T., Wicker T., Zeeman S.C., Santelia D. (2019). The evolution of functional complexity within the β-amylase gene family in land plants. BMC Evol. Biol..

[35] Rathore R.S., Garg N., Garg S., Kumar A. (2009). Starch phosphorylase: Role in starch metabolism and biotechnological applications. Crit. Rev. Biotechnol..

[36] Voll L.M., Hajirezaei M.R., Czogalla-Peter C., Lein W., Stitt M., Sonnewald U., Börnke F. (2009). Antisense inhibition of enolase strongly limits the metabolism of aromatic amino acids, but has only minor effects on respiration in leaves of transgenic tobacco plants. New Phytol..

[37] Schneider M., Knuesting J., Birkholz O., Heinisch J.J., Scheibe R. (2018). Cytosolic GAPDH as a redox-dependent regulator of energy metabolism. BMC Plant Biol..

