# Supplementary material for: Mango Fructokinases Inhibit Sugar Accumulation and Enhance Energy Metabolism in Transgenic Tomato
Source: Plants (Basel). 2025 Nov 19;14(22):3526. doi: 10.3390/plants14223526 (PMC12655981; doi:10.3390/plants14223526)
Supplement: Supplementary file 1 [file plants-14-03526-s001.zip › Figures.pdf]

```
R-MiFRK1    ATGGATATGAAAGCAGGATCTGGGAACAGAAATTCACTTGTCGTTTGCTTTGGGGAGATG
T-MiFRK1    ATGGATATGAAAGCAGGATCTGGGAACAGAAATTCACTTGTCGTTTGCTTTGGGGAGATG
            ************************************************************

R-MiFRK1    TTAATTGACTTTGTCCCAACAGTTGGAGGGGTTTCACTTGCTGAAGCACCAGCTTTCAAG
T-MiFRK1    TTAATTGACTTTGTCCCAACAGTTGGAGGGGTTTCACTTGCTGAAGCACCAGCTTTCAAG
            ************************************************************

R-MiFRK1    AAGGCTCCTGGTGGTGCTCCCGCTAATGTGGCTGTTGGGATATCAAGGCTCGGAGGTTCA
T-MiFRK1    AAGGCTCCTGGTGGTGCTCCCGCTAATGTGGCTGTTGGGATATCAAGGCTCGGAGGTTCA
            ************************************************************

R-MiFRK1    TCTGCCTTTGTTGGCAAGGTAGGTGATGATGAATTTGGTTACATGTTGGCTGACATTTTG
T-MiFRK1    TCTGCCTTTGTTGGCAAGGTAGGTGATGATGAATTTGGTTACATGTTGGCTGACATTTTG
            ************************************************************

R-MiFRK1    AAGCAAAACAATGTTGATACCTCTAGTGTGCGATATGATTCCTCTGCAAGGACTGCTTTG
T-MiFRK1    AAGCAAAACAATGTTGATACCTCTAGTGTGCGATATGATTCCTCTGCAAGGACTGCTTTG
            ************************************************************

R-MiFRK1    GCATTTGTTACACTCAGAGCTGATGGTGAACGTGAATTTCTGTTTTTTCGTCATCCAAGT
T-MiFRK1    GCATTTGTTACACTCAGAGCTGATGGTGAACGTGAATTTCTGTTTTTTCGTCATCCAAGT
            ************************************************************

R-MiFRK1    GCTGATATGCTTCTTCATGAATCAGAACTTGATAAAAAACTGATTAAGCAGGCCAGAATC
T-MiFRK1    GCTGATATGCTTCTTCATGAATCAGAACTTGATAAAAAACTGATTAAGCAGGCCAGAATC
            ************************************************************

R-MiFRK1    TTCCACTATGGTTCTATTAGTTTGATTGCAGAACCTTGCAAATCAACTCATCTTGCTGCC
T-MiFRK1    TTCCACTATGGTTCTATTAGTTTGATTGCAGAACCTTGCAAATCAACTCATCTTGCTGCC
            ************************************************************

R-MiFRK1    ATGAAAATAGCCAAAGAGTCTGGTAGCATCCTCTCTTATGATCCAAATTTGAGATTGCCG
T-MiFRK1    ATGAAAATAGCCAAAGAGTCTGGTAGCATCCTCTCTTATGATCCAAATTTGAGATTGCCG
            ************************************************************

R-MiFRK1    CTATGGCCATCAGAAGATGCTGCTCGGGAGGGCATAATGAGCATATGGGGTCAAGCAGAT
T-MiFRK1    CTATGGCCATCAGAAGATGCTGCTCGGGAGGGCATAATGAGCATATGGGGTCAAGCAGAT
            ************************************************************

R-MiFRK1    ATTATTAAGATAAGTGAGGATGAAATCACATTCTTAACTGGAGGTGATGATCATAATGAC
T-MiFRK1    ATTATTAAGATAAGTGAGGATGAAATCACATTCTTAACTGGAGGTGATGATCATAATGAC
            ************************************************************

R-MiFRK1    GATAATGTAGTGTTAGGGAAGCTGTTTCATCCTAATCTCAAGCTTTTGGTTGTAACTGAA
T-MiFRK1    GATAATGTAGTGTTAGGGAAGCTGTTTCATCCTAATCTCAAGCTTTTGGTTGTAACTGAA
            ************************************************************

R-MiFRK1    GGGTCTAAAGGTTGTAGATATTATACCAAGGAATTCAGAGGTTTTGTTCCTGGTGTTAAA
T-MiFRK1    GGGTCTAAAGGTTGTAGATATTATACCAAGGAATTCAGAGGTTTTGTTCCTGGTGTTAAA
            ************************************************************

R-MiFRK1    GCTATATCTGTGGACACCACTGGTGCTGGTGATGCATTTGTCAGTGGGATACTTAGCTGC
T-MiFRK1    GCTATATCTGTGGACACCACTGGTGCTGGTGATGCATTTGTCAGTGGGATACTTAGCTGC
            ************************************************************

R-MiFRK1    CTGGCTTCTGATCTAAGCCTGTTCAAGGACGAGAAGCGGCTTAGAGAAGCTCTACTTTTT
T-MiFRK1    CTGGCTTCTGATCTAAGCCTGTTCAAGGACGAGAAGCGGCTTAGAGAAGCTCTACTTTTT
            ************************************************************

R-MiFRK1    GCAAATGCCTGTGGTGCCCTCACAGTAACAGAGAGGGGTGCAATTCCTGCACTGCCTACA
T-MiFRK1    GCAAATGCCTGTGGTGCCCTCACAGTAACAGAGAGGGGTGCAATTCCTGCACTGCCTACA
            ************************************************************

R-MiFRK1    AAAGAAGCTGTCCTGAAACTCTTAGAACCAGTTGCTGCATCATAA
T-MiFRK1    AAAGAAGCTGTCCTGAAACTCTTAGAACCAGTTGCTGCATCATAA
            *********************************************
```

Figure S1. Comparison of *FRK1* cDNA sequences between 'Renong No.1' and 'Tainong No.1'. The asterisk (*) indicates identical cDNA sequence.

```
R-MiFRK2    ATGGCTTCAAACGGCGCAGTTTCCGGCTCCAGTCTCATCGTCTCCTTCGGGGAGATGCTG
T-MiFRK2    ATGGCTTCAAACGGCGCAGTTTCCGGCTCCAGTCTCATCGTCTCCTTCGGGGAGATGCTG
            ************************************************************

R-MiFRK2    ATCGACTTCGTGCCGACGGTATCCGGCGTCTCCCTCGCCGAGGCCCCCGGCTTCCTCAAG
T-MiFRK2    ATCGACTTCGTGCCGACGGTATCCGGCGTCTCCCTCTCCGAGGCTCCCGGCTTCCTCAAG
            ************************************ ******* ***************

R-MiFRK2    GCTCCGGGTGGCGCCCCGGCCAACGTTGCTATCGCTGTGGCTCGCCTTGGAGGCAAGGCA
T-MiFRK2    GCTCCTGGTGGCGCCCCGGCCAACGTTGCTATCGCTGTGGCTCGCCTCGGAGGCAAGGCA
            ***** ***************************************** ************

R-MiFRK2    GCCTTCGTCGGCAAACTCGGCGACGATGAGTTCGGCCACATGTTGGCCGGGATCTTGAAG
T-MiFRK2    GCCTTCGTCGGCAAACTCGGCGACGATGAGTTCGGCCACATGTTGGCCGGGATCTTGAAG
            ************************************************************

R-MiFRK2    GAGAATGGAGTCAGCGGCGCCGGCATCAACTTTGACCAAGGCGCCAGGACTGCTCTCGCC
T-MiFRK2    GAGAATGGAGTCAGCGGCGACGGCATCAACTTTGACCAAGGCGCCAGGACTGCTCTCGCC
            ******************* ****************************************

R-MiFRK2    TTCGTGACTCTACGCGCCGATGGTGAGCGTGAGTTCATGTTTTATAGGAATCCCAGTGCT
T-MiFRK2    TTCGTGACTCTACGCGCTGATGGTGAGCGTGAGTTCATGTTTTATAGGAATCCCAGTGCT
            ***************** ******************************************

R-MiFRK2    GACATGCTGTTGAGACCTGACGAATTGAATCTGGAGCTTATCAAATCCGCCAAGGTCTTC
T-MiFRK2    GATATGCTGTTGAGACCTGACGAGTTGAATCTGGAGCTTATCAAATCCGCCAAGATCTTC
            ** ******************** ****************************** *****

R-MiFRK2    CATTATGGATCAATAAGTTTGATCGTGGAGCCATGTAGATCAGCTCACTTAAAGGCAATG
T-MiFRK2    CATTATGGATCAATAAGTTTGATCGTGGAGCCATGTAGATCAGCTCACTTAAAGGCAATG
            ************************************************************

R-MiFRK2    GAGGTTGCTAAGGATGCAGGAGCCCTCCTTTCCTACGACCCAAACCTCAGACTACCATTG
T-MiFRK2    GAGGTTGCTAAGGATGCAGGAGCCCTCCTTTCCTACGACCCAAACCTCAGACTACCATTG
            ************************************************************

R-MiFRK2    TGGCCCTCTCCCGAGGAGGCGCGCACACAGATCCTGAGCATTTGGGACAAGGCAGAGGTG
T-MiFRK2    TGGCCCTCTCCCGAGGAGGCGCGCACACAGATCCTGAGCATTTGGGACAAGGCAGAGGTG
            ************************************************************

R-MiFRK2    ATCAAGGTCAGTGATGTGGAGCTGGAGTTCCTAACTGGTAGTGACAAGATTGATGACGAA
T-MiFRK2    ATCAAGGTCAGTGATGTGGAGCTGGAGTTCCTAACTGGTAGTGACAAGATTGATGACGAA
            ************************************************************

R-MiFRK2    TCTGCCTTGTCGCTATGGCATCCTAACCTGAAGCTCCTTTTGGTCACTCTTGGTGAAAAG
T-MiFRK2    TCTGCCTTGTCGCTATGGCATCCTAACCTGAAGCTCCTTTTGGTCACTCTTGGTGAAAAG
            ************************************************************

R-MiFRK2    GGTTGTAGGTACTATACTAAGGGTTTCAAAGGAGAAGTGGAAGCCTTCCATGTCAACACA
T-MiFRK2    GGTTGTAGGTACTATACTAAGGGTTTCAAAGGAGCAGTGGAAGCCTTCCATGTCAACACA
            ********************************** *************************

R-MiFRK2    GTAGATACAACTGGTGCTGGCGATGCATTTGTCGGTGCTTTACTATGCAATATTGTCGAT
T-MiFRK2    GTAGATACAACTGGTGCTGGCGATGCATTTGTCGGTGCTTTACTATGCAAGATTGTCGAT
            ************************************************** *********

R-MiFRK2    GATCACTCCCTTCTTGAGGACGAAGCAAGGCTGAGAAAAGTACTTAAATATGCCAATGCA
T-MiFRK2    GATCACTCCCTTCTTGAGGACGAAGCAAGGCTGAGAGAAGTACTTAAATATGCCAATGCA
            ************************************ ***********************

R-MiFRK2    TGTGGAGCCATAACCACCACCAAAAAGGGAGCCATTCCAGCTCTTCCCAAAGAGGCTGAT
T-MiFRK2    TGTGGAGCCATAACCACCACCAAAAAGGGAGCCATTCCAGCTCTTCCCAGAGAGGCTGAT
            ************************************************* **********

R-MiFRK2    GTCCTCAGCTTAATCAAAGGGGCATATTAA
T-MiFRK2    GTCCTCAGCTTAATCAAAGGGGCATATTAA
            ******************************
```

Figure S2. Comparison of *FRK2* cDNA sequences between 'Renong No.1' and 'Tainong No.1'. The asterisk (*) indicates identical cDNA sequence.

```
R-MiFRK1   MDMKAGSGNRNSLVVCFGEMLIDFVPTVGGVSLAEAPAFKKAPGGAPANVAVGISRLGGS
T-MiFRK1   MDMKAGSGNRNSLVVCFGEMLIDFVPTVGGVSLAEAPAFKKAPGGAPANVAVGISRLGGS
           ************************************************************

R-MiFRK1   SAFVGKVGDDEFGYMLADILKQNNVDTSSVRYDSSARTALAFVTLRADGEREFLFFRHPS
T-MiFRK1   SAFVGKVGDDEFGYMLADILKQNNVDTSSVRYDSSARTALAFVTLRADGEREFLFFRHPS
           ************************************************************

R-MiFRK1   ADMLLHESELDKKLIKQARIFHYGSISLIAEPCKSTHLAAMKIAKESGSILSYDPNLRLP
T-MiFRK1   ADMLLHESELDKKLIKQARIFHYGSISLIAEPCKSTHLAAMKIAKESGSILSYDPNLRLP
           ************************************************************

R-MiFRK1   LWPSEDAAREGIMSIWGQADIIKISEDEITFLTGGDDHNDDNVVLGKLFHPNLKLLVVTE
T-MiFRK1   LWPSEDAAREGIMSIWGQADIIKISEDEITFLTGGDDHNDDNVVLGKLFHPNLKLLVVTE
           ************************************************************

R-MiFRK1   GSKGCRYYTKEFRGFVPGVKAISVDTTGAGDAFVSGILSCLASDLSLFKDEKRLREALLF
T-MiFRK1   GSKGCRYYTKEFRGFVPGVKAISVDTTGAGDAFVSGILSCLASDLSLFKDEKRLREALLF
           ************************************************************

R-MiFRK1   ANACGALTVTERGAIPALPTKEAVLKLLEPVAAS
T-MiFRK1   ANACGALTVTERGAIPALPTKEAVLKLLEPVAAS
           **********************************

R-MiFRK2   MASNGAVSGSSLIVSFGEMLIDFVPTVSGVSLAEAPGFLKAPGGAPANVAIAVARLGGKA
T-MiFRK2   MASNGAVSGSSLIVSFGEMLIDFVPTVSGVSLSEAPGFLKAPGGAPANVAIAVARLGGKA
           ******************************** ***************************

R-MiFRK2   AFVGKLGDDEFGHMLAGILKENGVSGAGINFDQGARTALAFVTLRADGEREFMFYRNPSA
T-MiFRK2   AFVGKLGDDEFGHMLAGILKENGVSGDGINFDQGARTALAFVTLRADGEREFMFYRNPSA
           ************************** *********************************

R-MiFRK2   DMLLRPDELNLELIKSAKVFHYGSISLIVEPCRSAHLKAMEVAKDAGALLSYDPNLRLPL
T-MiFRK2   DMLLRPDELNLELIKSAKIFHYGSISLIVEPCRSAHLKAMEVAKDAGALLSYDPNLRLPL
           ****************** *****************************************

R-MiFRK2   WPSPEEARTQILSIWDKAEVIKVSDVELEFLTGSDKIDDESALSLWHPNLKLLLVTLGEK
T-MiFRK2   WPSPEEARTQILSIWDKAEVIKVSDVELEFLTGSDKIDDESALSLWHPNLKLLLVTLGEK
           ************************************************************

R-MiFRK2   GCRYYTKGFKGEVEAFHVNTVDTTGAGDAFVGALLCNIVDDHSLLEDEARLRKVLKYANA
T-MiFRK2   GCRYYTKGFKGAVEAFHVNTVDTTGAGDAFVGALLCKIVDDHSLLEDEARLREVLKYANA
           *********** ************************ *************** *******

R-MiFRK2   CGAITTTKKGAIPALPKEADVLSLIKGAY
T-MiFRK2   CGAITTTKKGAIPALPREADVLSLIKGAY
           **************** ************
```

Figure S3. Comparison of FRK1 and FRK2 protein sequences between 'Renong No.1' and 'Tainong No.1. The asterisk (*) indicates identical amino acid sequence.

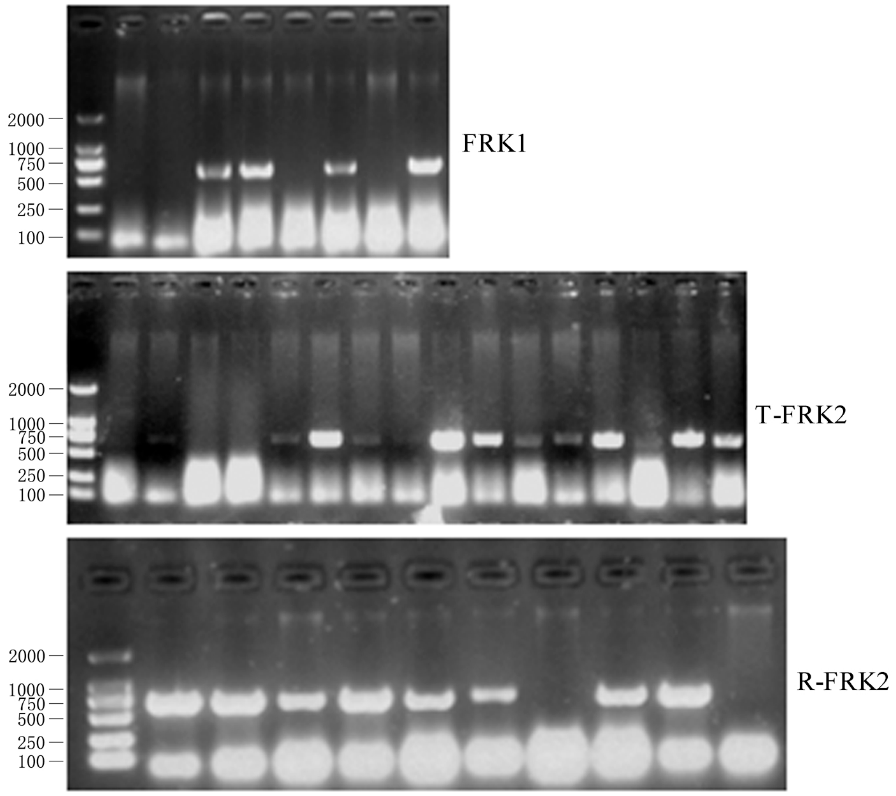

Figure S4. PCR validation of resistant strain lines.

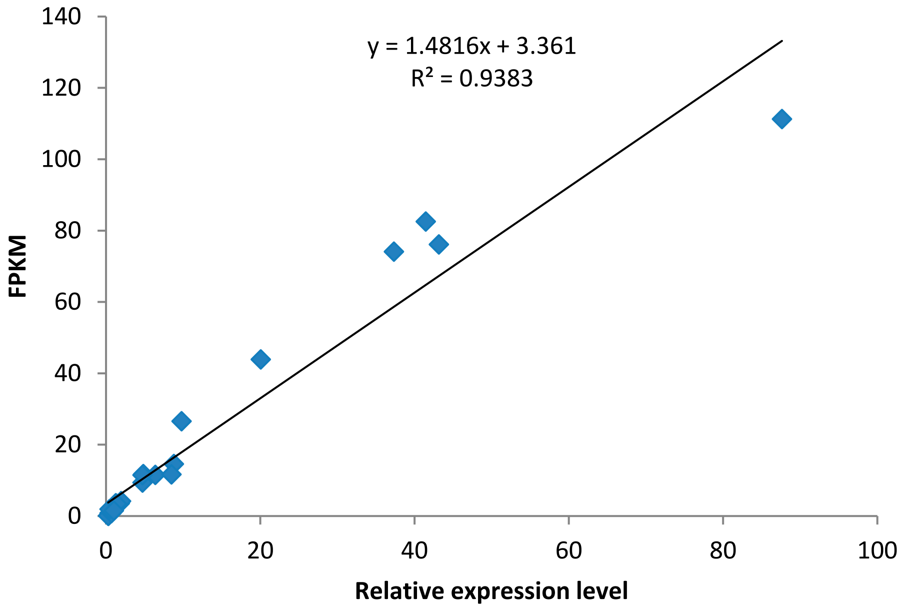

Figure S5. Linear correlation between relative expression level and FPKM value.
