# Supplementary material for: Mango Fructokinases Inhibit Sugar Accumulation and Enhance Energy Metabolism in Transgenic Tomato
Source: Plants (Basel). 2025 Nov 19;14(22):3526. doi: 10.3390/plants14223526 (PMC12655981; doi:10.3390/plants14223526)
Supplement: Supplementary file 1 [file plants-14-03526-s001.zip › Table S1.pdf]

Table S1. Name, sequence, and application of the primers employed in this study.

Name	Sequence	Application
MiFRK1 F1	CGCGGATCCATGGATATGAAAGCAGGAT	constructing
MiFRK1 R1	ACGCGTCGACAGTTGCAGCAACTGGTTCT	overexpression vectors
MiFRK2 F1	CGCGGATCCATGGCTTCAAACGGCGCAG	constructing
MiFRK2 R1	ACGCGTCGACATATGCCCCTTTGATTAAG	overexpression vectors
NPT II -F	GTGGAGAGGCTATTCGGCTATGACTG	identification of positive
NPT II -R	AGCTCTTCAGCAATATCACGGGTAGC	transgenic tomato
ENO-F	TGGTGCGATAAAGACG	qPCR confirmed the gene
ENO-R	CTACTACCGTGGACAT	expression level
SP-F	GACGCTAAACTACCCTG	qPCR confirmed the gene
SP-R	CCATTCCTTTGACCC	expression level
ACT-F	GTCCTCTTCCAGCCATCCAT	qPCR reference gene
ACT-R	ACCACTGAGCACAATGTTACCG	primers
